# Reduction of metal adducts in oligonucleotide mass spectra in ion‐pair reversed‐phase chromatography/mass spectrometry analysis

**DOI:** 10.1002/rcm.7596

**Published:** 2016-06-22

**Authors:** Robert E. Birdsall, Martin Gilar, Henry Shion, Ying Qing Yu, Weibin Chen

**Affiliations:** ^1^ Waters Corp.34 Maple StMilfordMA01757‐3604USA

## Abstract

**Rationale:**

Electrospray ionization mass spectrometry (ESI‐MS)‐based techniques commonly used in oligonucleotide analyses are known to be sensitive to alkali metal adduct formation. Adducts directly impact the sensitivity of MS‐based analyses as the available charge is distributed across the parent peak and adduct(s). The current study systematically evaluated common liquid chromatography (LC) components in LC/ESI‐MS configurations used in oligonucleotide analysis to identify metal adduct contributions from LC instrumentation.

**Methods:**

A UPLC liquid chromatography system was configured with a single quadrupole MS detector (ACQUITY QDa, Waters Corp.) to monitor adduct formation in oligonucleotide separations. An ion‐pairing mobile phase comprised of 15 mM triethylamine and 400 mM hexafluoro‐2‐propanol was used in conjunction with an oligonucleotide separation column (Waters OST BEH C18, 2.1 mm × 50 mm) for all separations. A 10‐min method was used to provide statistical figures of merit and evaluate adduct formation over time.

**Results:**

Trace alkali metal salts in the mobile phase and reagents were determined to be the main source of metal salt adducts in LC/ESI‐MS‐based configurations. Non‐specific adsorption sites located throughout the fluidic path contribute to adduct formation in oligonucleotide analyses. Ion‐pairing mobile phases prepared at neutral or slightly basic pH result in up to a 57% loss of spectral abundance to adduct formation in the current study.

**Conclusions:**

Implementation of a short low pH reconditioning step was observed to effectively displace trace metal salts non‐specifically adsorbed to surfaces in the fluidic path and was able to maintain an average MS spectral abundance ≥94% with a high degree of repeatability (relative standard deviation (R.S.D.) 0.8%) over an extended time study. The proposed method offers the ability to rapidly regenerate adsorption sites with minimal impact on productivity while retaining assay sensitivity afforded by MS detection with reduced adduct formation. © 2016 The Authors. *Rapid Communications in Mass Spectrometry* Published by John Wiley & Sons Ltd.

Research into therapeutic oligonucleotides has received steadily increasing attention from the pharmaceutical industry due to potential applications using deoxyribonucleic acid (DNA) sense/antisense oligonucleotides, interfering ribonucleic acid (RNAi)‐based therapies, and nucleic acid based aptamers.^1^, ^2^, ^3^, ^4^, ^5^ This renewed interest has in part been fueled by the commercialization of therapeutic oligonucleotides such as Formivirsen^6^ and Pegaptanib^7^ in the treatment of cytomegalovirus retinitis and age‐related macular degeneration, respectively.

Therapeutic oligonucleotides are produced synthetically from their corresponding nucleotides, typically ranging between 15 nucleotides (15'mer or 15 nt) and 30 nucleotides (30'mer or 30 nt) although oligonucleotides with lengths of greater than 30 nt have also gathered increasing interest from the pharmaceutical industry.^1^ Chemical modifications to the phosphodiester backbone are commonly incorporated into synthetic oligonucleotides to increase stability and safety *in vivo* against endo‐ and exonucleases as well as improve efficacy through increased cellular uptake and binding properties.^1^, ^8^, ^9^ While the synthesis process of oligonucleotides is well controlled, several factors can alter the end product. Quality of starting materials can impact the purity of products while random insertion/deletions can result in improper sequence generation.^10^, ^11^, ^12^ Furthermore, synthetic errors (undesired nucleotide order switches) and phosphate backbone modification producing chiral centers result in difficult to separate isomers and diastereomers, respectively.^13^ With over 100 therapeutic oligonucleotides currently in development or in clinical trials, factors such as safety, efficacy, and stability are leading concerns for pharmaceutical companies and regulatory agencies.^1^ To this end, characterization methods that are robust, quantitative, and offer adequate selectivity are highly desirable in the analysis of therapeutic oligonucleotides.

Due to the negatively charged phosphorodiester backbone the chromatographic methods of choice for oligonucleotide characterization are ion‐exchange chromatography (IEC) and ion‐pairing reversed‐phase chromatography (IP‐RPLC).^14^, ^15^, ^16^, ^17^, ^18^, ^19^ Charge‐based separations such as anion exchangers are well suited in the characterization of N‐X deletions; however, depurination events, base inversion isomers, and other base modifications are not readily characterized using IEC.^1^ Furthermore, buffers and salt gradients typically used in IEC prevent coupling to mass spectrometry (MS) as a complementary orthogonal technique for the characterization of oligonucleotides when dealing with challenging base modifications. Techniques such as IP‐RPLC have become prevalent in the characterization of oligonucleotides in part due to their compatibility with MS‐based techniques as demonstrated by Apffel and colleagues using the IP base triethylamine (TEA) buffered in hexafluoroisopropanol (HFIP).^18^, ^20^ When adsorbed onto hydrophobic bonded phases, IP reagents such as n‐alkylamines provide a means to separate oligonucleotides with high separation efficiency based on charge interaction of the phosphodiester backbone and to a lesser degree the secondary structure of the oligonucleotide and hydrophobicity of the base nucleotides.^11^, ^17^, ^21^, ^22^ In addition to providing accurate mass information for the characterization of oligonucleotides, MS‐based methods are highly desirable in oligonucleotide assays that require sensitive techniques such as toxicological profiles, metabolite studies, and determination of pharmacodynamics/pharmacokinetic parameters.^14^, ^17^, ^23^, ^24^, ^25^ The merits of increased sensitivity and specificity afforded by MS‐based techniques are well established, but are not without their own challenges.^12^, ^26^, ^27^, ^28^, ^29^, ^30^, ^31^

Electrospray ionization (ESI)‐MS‐based techniques commonly used in oligonucleotide analyses are known to be sensitive to alkali metal adduct formation.^18^, ^23^, ^32^, ^33^, ^34^ Positively charged cations of alkali metal salts such as sodium (Na^+^) and potassium (K^+^) are electrostatically attracted to the negatively charged polyanionic backbone of oligonucleotides.^35^, ^36^ Alkali metal adducts, which can occur singly, multiply, or as combinations of single and multiple charges, directly impact the sensitivity of MS‐based analyses as the available charge is distributed across the parent peak and its adducts. This problem is further compounded with longer oligonucleotides as sequence length, number of observed charge states, and base modifications can impact the degree of adduct formation and spectral complexity.^37^, ^38^ Strategies in the mitigation of alkali metal adducts in the analysis of oligonucleotides using ESI‐MS‐based approaches have included offline and online desalting procedures with varying success.

Offline desalting procedures that incorporate hydrophobic resins, molecular weight cutoff filters, and solid‐phase extraction techniques have been shown to be effective in reducing adduct formation of oligonucleotides in ESI‐MS‐based methods.^10^, ^39^, ^40^, ^41^, ^42^ Nonetheless, the additional sample preparation steps required are not readily amendable to high‐throughput platforms. Online desalting strategies have included incorporation of microdialysis or cation‐exchange chromatography.^43^, ^44^ These techniques, while more amenable to high‐throughput methods, can increase instrument configuration complexity and require additional method re‐conditioning/equilibration steps which can impact productivity. A more appealing alternative to reducing alkali metal adducts in oligonucleotide analyses has been to use the IP‐RPLC column itself to desalt samples. Oberacher *et al.* successfully demonstrated analysis of nucleic acids solutions containing up to 1.7 mol/L sodium chloride with reduced adducts using an MS‐based technique. Optimized chromatography conditions including column temperature were cited as contributing factors in adduct mitigation.^12^ Alternatively, sample additives that act as cation scavengers to suppress adduct formation via displacement mechanisms have also been shown to be successful in reducing adducts. Limbach and colleagues observed that the addition of *trans*‐1,2‐cyclohexanediaminetetraacetic acid monohydrate (CDTA), a metal chelator, reduced adduct formation in the analysis of RNA.^45^ Alternatively, addition of bases such as piperidine or TEA as observed by Grieg and Griffey as well as others was found to suppress adduct formation.^35^, ^36^, ^46^ In contrast to Limbach *et al*., an extensive study by Gong and McCullagh of IP reagents buffered with HFIP found that metal chelators such as CDTA and ethylenediaminetetraacetic acid (EDTA) did not have a significant impact on adduct formation.^38^ Their work indicated that adduct formation was dependent on oligonucleotide size. Interestingly, with the exception of the 10 nt polyT sequence, more than 25% of the MS signal was in a metal adduct form. Despite these conflicting reports, the relevance of suppressing cation adduction is evident in the diversity of strategies employed across instrument configurations and experimental settings.

These approaches, while effective in reducing adduct formation, neglect to address LC instrument contributions to metal salt adducts, a challenging task considering the pervasive nature of alkali metal salts in LC separations.^28^, ^29^ Glass surfaces such as the ones found in reservoir bottles as well as sample vials can contain trace alkali metal salts as a byproduct of the process used to manufacture them. Leaching of these trace metal salts can occur when used in the presence of solvents, acids, and bases.^47^ The contamination of the solvents from preparation components (e.g. make‐up water, additives, and buffer components) used in chromatographic separations can also increase the concentration of salt ions present in an analytical separation. Similarly, the abundance of alkali metal salts in biological samples can also contribute to adduct formation in ESI‐MS‐based analyses.^12^, ^48^ Furthermore, the chromatography system itself can also act as a source of metal adduct ions as alkali metal salts are deposited on high surface area points of contact found throughout the system such as mixers, filtering frits, and column frits. A root‐cause study of LC instrument contributions to common metal adducts observed in LC/ESI‐MS‐based oligonucleotide analyses appears to be absent from the current body of literature.

The objective of this study is to systematically evaluate contributing factors in metal adduct formation in IP‐RPLC analysis of oligonucleotides when coupled to ESI‐MS from an LC instrument perspective. From these findings strategies to mitigate alkali metal adducts in oligonucleotide analyses with minimal impact on methodology are discussed.

## Experimental

### Materials

Triethylamine (P/N 90337, ≥99.5% purity) and 1,1,1,3,3,3‐hexafluoro‐2‐propanol manufactured by Aldrich Chemicals (P/N 105228, ≥99% purity) as well as triethylamine (P/N 65897, ≥99.5% purity) and 1,1,1,3,3,3‐hexafluoro‐2‐propanol manufactured by Fluka (P/N 42060, ≥99.8% purity) were purchased from Sigma Aldrich (St. Louis, MO, USA). LC‐UV grade solvents (Optima series) and 500 mL low‐density polyethylene bottles were purchased from Fisher Scientific (Pittsburg, PA, USA). LC/MS‐grade solvents (CHROMASOLV series) were purchased from Sigma Aldrich. Polypropylene screw neck vials with cap (12 × 32 mm) were purchased from Waters Corp. (Milford, MA, USA). DNA sequencing grade phosphodiester ssRNA sequences with double thymine overhangs were ordered from Integrated DNA Technology (Coralville, IA, USA) with the sequence 5′‐UCGUCAAGCGAUUACAAGGTT‐3′ (strand 1) and 5′‐TTCCUUGUAAUCGCUUGACGA‐3′ (strand 2). Stock oligonucleotide samples were prepared at a concentration of 100 μmol in their received vials using MS‐grade water. Working samples were prepared at a concentration of 10 pmol/μL in LoBind Eppendorf tubes (Hauppauge, NY, USA) using MS‐grade water prior to transfer to polypropylene sample vials. Mass loads on column were kept constant at 50 pmol or 5 μL injections.

### Chromatography

A UPLC liquid chromatography system configured with Bio‐inert tubing (ACQUITY H‐Class Bio, Waters Corp.) was used for the study. A tunable UV detector (ACQUITY TUV, Waters Corp.) equipped with a 5‐mm titanium flow cell was used for optical detection. Single wavelength detection was performed at an A_max_ of 260 nm with a sampling rate of 20 Hz. An OST BEH C18 column (130 Å, 1.7 μm, 2.1 mm × 50 mm; Waters Corp.) was used for all separations at a set temperature of 60°C. Mobile phases (MPs) were prepared gravimetrically as MP A: 15 mM TEA, 400 mM HFIP in H_2_O; MP B: 15 mM TEA, 400 mM HFIP in methanol; MP C: H_2_O, 0.2% formic acid (FA) v/v, MP D: MeOH. High pH reconditioning gradients using MP A and MP B were performed as shown in Table 1. Low pH reconditioning gradients using MP A, MP B, MP C, and MP D were performed to regenerate the surfaces throughout the fluidic path as shown in Table 1.

**Table 1:** Chromatographic separation gradients. High pH and low pH reconditioning gradients used in the separation of oligonucleotides

Time (min)	Flow (mL/min)	%A	%B	%C	%D
High pH Reconditioning Gradient					
0.00	0.200	82.0	18.0	0.0	0.0
4.00	0.200	80.0	20.0	0.0	0.0
4.01	0.200	50.0	50.0	0.0	0.0
6.01	0.200	50.0	50.0	0.0	0.0
6.02	0.200	82.0	18.0	0.0	0.0
10.00	0.200	82.0	18.0	0.0	0.0
Low pH Reconditioning Gradient					
0.00	0.200	82.0	18.0	0.0	0.0
4.00	0.200	80.0	20.0	0.0	0.0
4.01	0.200	0.0	0.0	50.0	50.0
5.01	0.200	0.0	0.0	50.0	50.0
5.02	0.200	82.0	18.0	0.0	0.0
10.00	0.200	82.0	18.0	0.0	0.0

### MS settings

A single quadrupole mass spectrometer (ACQUITY QDa, Waters Corp.) was used for MS analysis post TUV detection to monitor adduct formation. The adduct formation in this study was also confirmed with high‐resolution MS instrumentation with expanded scan range capabilities as needed. MS data was collected throughout the separation as defined in the chromatography section with the flow continuously passing through the MS capillary and the MS polarity mode set to negative. Adjustable instrument settings were set as follows: capillary voltage 0.8 kV, sample cone 20.0 V, source temperature 600°C. An *m*/*z* scan range was collected from 410 to 1250 *m*/*z*. MS data was processed within the chromatography data system MassLynx (Waters Corp.) using an equal number of scans to assess alkali metal adduct formation using the MaxEnt 1 algorithm for deconvolution (resolution =0.7, peak width = 1). High‐throughput screening MS acquisition data was processed with ProMass (Novatia LLC, PA, USA) using default parameters.

## Results and Discussion

### Borosilicate glassware

Efforts to minimize alkali metal salt adduct formation were taken prior to beginning the current study. As a potential point source of metal salt ions, solvent glassware and sample vials were replaced with plastic alternatives constructed from polyethylene and polypropylene, respectively. Solvent bottles were soaked overnight in 60% isopropyl alcohol to remove leachable impurities such as residual monomer and hardening agents.^49^ Polypropylene sample vials were used as received. To establish a baseline response the LC system was purged with a 30% phosphoric acid solution to wash out residual alkali metal salts in the fluidic path followed immediately by rinsing with MS‐grade water. After cleaning the LC system was prepared for oligonucleotide analysis using a TEA/HFIP IP‐RPLC mobile phase prepared with the UHPLC‐UV‐grade solvents. A 10‐min method was used to provide statistical figures of merit and evaluate adduct formation over time.

**Figure 1:**
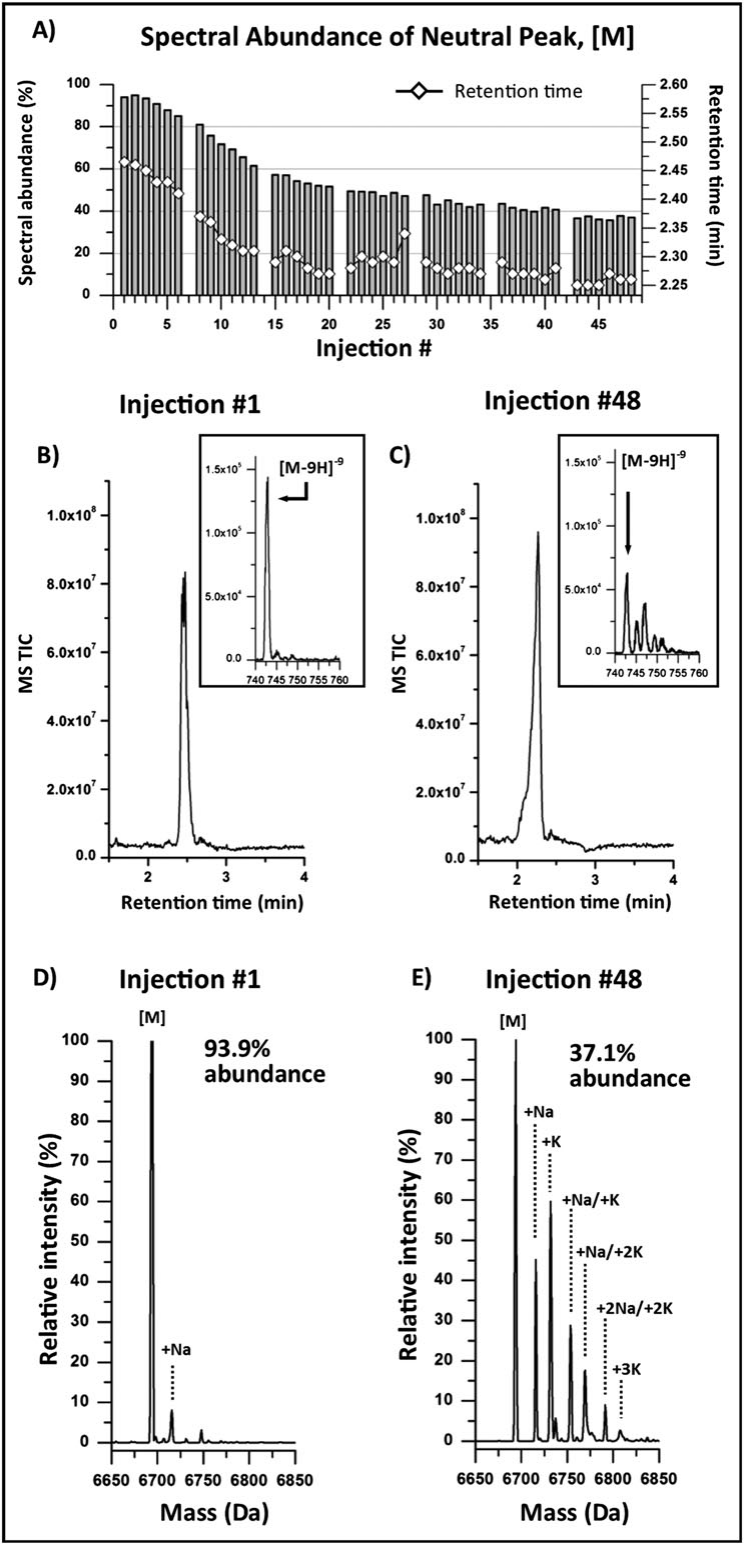
IP‐RPLC/MS assessment of oligonucleotide strand 1. (A) Deconvoluted peak intensity for the neutral parent peak [M] of the ssRNA strand 1 as percent total intensity was used to determine spectral abundance over the course of an 8‐h injection series. Oligonucleotide retention time was observed to shift from (B) 2.45 min to (C) 2.26 min across the injection series. (D) Deconvoluted spectrum of Injection 1 was used as a baseline reference with the neutral peak [M] being observed at 93.9% spectral abundance when compared to the +Na adduct form (6.1%) in a clean LC system. (E) Injection 48 indicates over 56.8% of the spectral abundance of the ssRNA was observed to be in an adduct form representing a contaminated system.

Using an injection series that incorporates a full 48‐well sample plate, ssRNA (strand 1) samples were prepared in MS‐grade water and transferred to polypropylene vials across the 48 wells. A different ssRNA (strand 2) was loaded in every 7^th^ vial position as a negative control for ProMass confirmation. A single quadrupole mass detector was configured in a serial configuration post‐optical detection to evaluate alkali metal salt adducts in the deconvoluted spectrum. As shown in Fig. 1(A), the spectral abundance of the deconvoluted ssRNA neutral peak [M] as a percentage of total peak intensity, including adduct forms, was plotted from the deconvoluted MS data over the course of the injection series. Figures 1(B) and 1(C) show the corresponding mass chromatogram and raw spectrum (inset) for the first and last injection, respectively. Injection 1 as shown in Fig. 1(D) was used as baseline reference for adduct evaluation. A sodium adduct was observed with a relative intensity of 6.1% with respect to the neutral peak. Considering the use of alkali metal salts in LC‐based methods, it is not unexpected that trace‐level impurities will still be present despite running the assay immediately following the system cleaning protocol; therefore, these levels were considered acceptable. The significant decrease in spectral abundance from 94% to 37% over the course of the 8‐h injection series (Fig. 1(A)) was unexpected in light of the fact the injection series was performed with a newly prepared set of TEA/HFIP buffers in a system configuration where plastic substitutes were used in lieu of borosilicate glassware. As shown in Fig. 1(E), using the deconvoluted MS data, the adduct forms were determined to be single and multiple adducts of sodium and potassium, confirmation of which was performed using the same LC configuration coupled to a high‐resolution MS instrument (Xevo G2‐XS QTof, Waters). Similar adduct profiles were observed in an identical experiment using borosilicate glassware in lieu of plastic alternatives (data not shown). This evidence suggests that borosilicate glassware is not a primary source of alkali metal salts when taking proper steps to clean glassware and use newly prepared mobile phases.

### Sample purity

**Figure 2:**
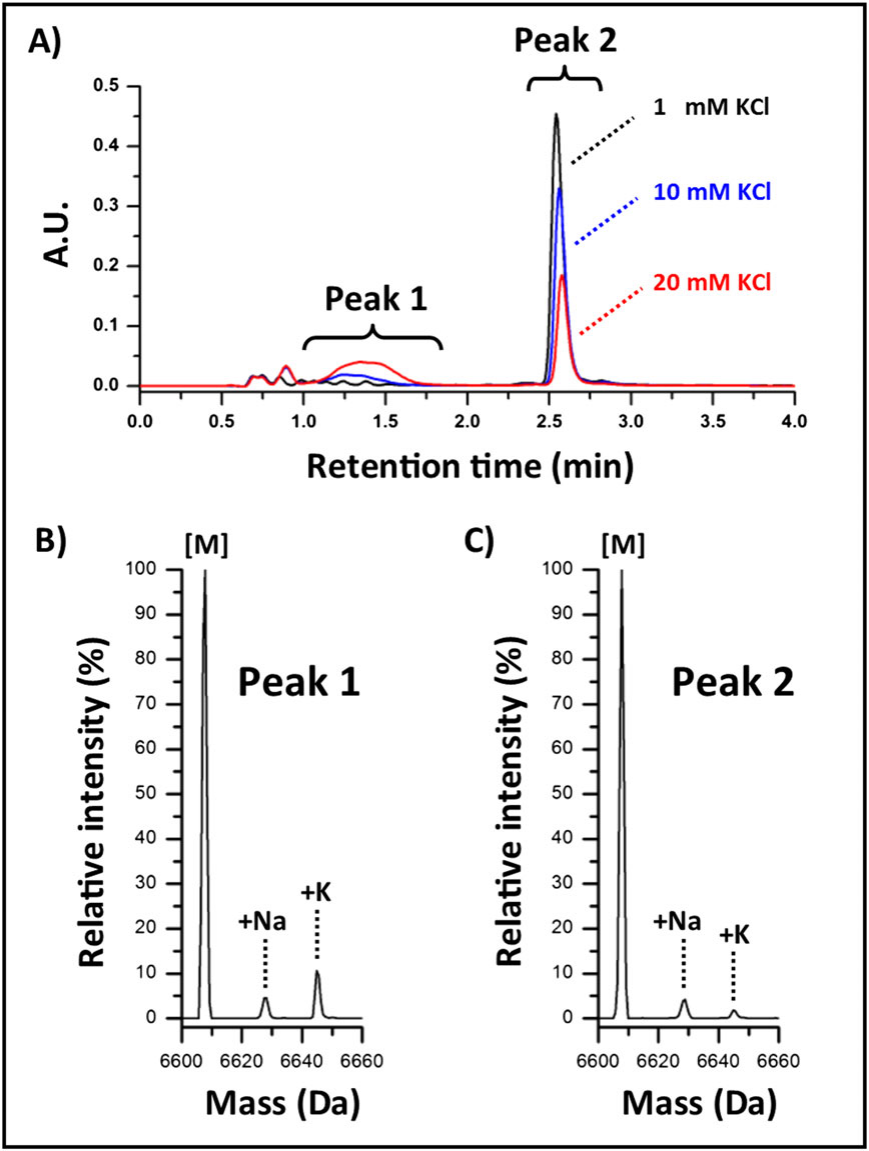
Sample salt concentration. (A) High‐throughput UV chromatograms of the ssRNA strand 2 prepared in 1 mM (black trace), 10 mM (blue trace), and 20 mM KCl (red trace). (B) Relative intensities of the +Na and +K adducts were observed to be 4.6% and 10.6%, respectively, for the deconvoluted spectrum of peak 1 from the 20 mM KCl sample. (C) Relative intensities of the +Na and +K adducts were observed to be 4.1% and 1.8%, respectively, for the deconvoluted spectrum of the later eluting peak 2 from the 20 mM KCl sample.

A spiking study was performed to evaluate sample salt concentration impacts on chromatography using the same system configuration with polyethylene mobile phase bottle and polypropylene sample vials. Prior to sample evaluation, the systems fluidic path was cleaned as before to remove residual metal salts. Sodium adducts were observed with a relative intensity of 5% using the ssRNA strand 2 sample prepared in water as a control to evaluate system cleanliness (data not shown). Potassium adducts were not observed in the control sample. A 100 mM KCl solution was used to prepare test samples of the ssRNA with a final salt concentration ranging from 0.5 mM KCl up to 50 mM KCl. Using the same LC configuration and 10‐min separation method as before, injection volumes were adjusted to maintain a 50 pmol on‐column mass load. Chromatogram overlays of samples prepared in 1 mM KCl, 10 mM KCl, and 20 mM KCl are shown in Fig. 2(A). Two peaks of interest were observed to change with increasing KCl concentration. The acquired MS data was investigated using the 20 mM KCl sample for insight into the behavior observed in the UV chromatograms.

MS spectra were combined using an equal number of scans from 1.0 to 2.0 min and 2.4 to 3.4 min for peak 1 and peak 2, respectively. As shown in Fig. 2(B), deconvolution of the peak 1 spectrum indicated the broad peak was predominantly the ssRNA with sodium and potassium adducts observed with relative intensities of 4.6% and 10.6%, respectively. Deconvolution of the peak 2 spectrum (as shown in Fig. 2(C)) indicated the peak was the ssRNA as well but with sodium and potassium adducts observed with lower relative intensities of 4.1% and 1.8%, respectively. The peak splitting observed in the UV chromatogram suggests that salts, when present in samples at elevated concentrations, can severely disrupt the ion‐pairing equilibrium and interfere with the charged‐based ion‐pairing retention mechanism. In addition, the observed decreasing adduct intensity from the MS data for the later eluting peak suggests that the samples are being simultaneously desalted as they are more retained on the RP column relative to KCl and other salts.^10^, ^12^ This observation was confirmed with no potassium adducts observed in the chromatographic profile of the ssRNA prepared in 50 mM KCl using a 30‐min gradient with a lower initial organic composition (MP B 5%) for improved IP efficiency. Collectively, this data suggests the samples, which were desalted post‐synthesis by the manufacturer in combination with the small injection volume (≤10 μL) being significantly diluted upon introduction into the eluent stream, are not a significant contributing factor to adduct formation in the experimental design of the current studies.

### Column salt tolerance

To evaluate column tolerance to metal salt exposure an isocratic method was designed with the MP composition of B set to 19% to allow the ssRNA strand 2 to elute within 1.5 min. Performing the assay using isocratic conditions minimizes the impact that the column reconditioning step may have in reducing adduct formation. The system was cleaned of residual alkali metal salts prior to the experiment. Using a water blank containing 100 mM KCl, 10‐μL injections were made with the isocratic method using a 1‐min run time. The ssRNA was prepared in water and injected after 0, 10, and 30 injections of the 100 mM KCl water blank using the same isocratic method with a 4‐min run time to allow sufficient time for the sample to elute.

**Figure 3:**
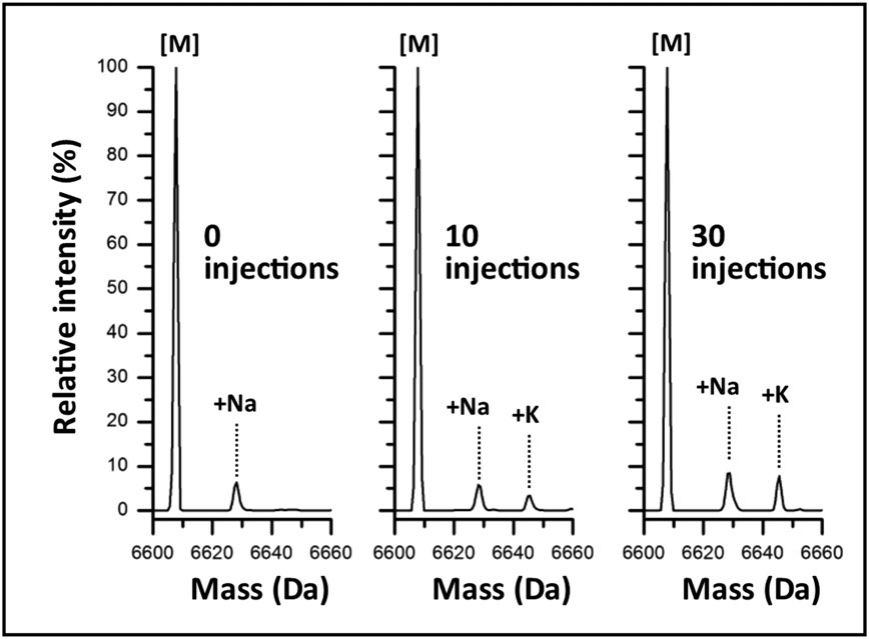
Column salt tolerance. Deconvoluted spectrum of the ssRNA strand 2 after 0, 10, and 30 injections of a water blank containing 100 mM KCl. Relative intensity of the sodium adduct was determined to be 6.3%, 5.8%, and 8.5% for the 0, 10, and 30 injection runs, respectively. The potassium adduct was not observable in the 0 injection run but was observed to increase proportionally in the 10 and 30 injection runs with relative intensity of 3.3% and 7.8%, respectively.

As seen in the deconvoluted MS spectrum shown in Fig. 3, adduct formation and intensity were observed to increase with multiple injections of the 100 mM KCl water blank. The baseline reference acquired prior to the salt blank injections (0 injections) indicated sodium adducts were present at a relative peak intensity of 6.3%, which was in line with previous experiments. The sodium adducts were observed to show a modest increase in relative peak intensity to 8.5% after the 30^th^ injection of the 100 mM KCl salt blank. Similar behavior was also observed with the potassium adducts, which increased proportionally in relative intensity from 3.3% to 7.8% following 10 and 30 KCl injections, respectively. The observation of metal adduct formation following injections of KCl blanks from this experiment suggests there is a degree of non‐specific adsorption of alkali metal ions occurring between the time of injection and detection, possibly in the column itself. This behavior was also observed in the borosilicate experiment when comparing the spectral abundance plots to the corresponding retention time of the ssRNA as shown in Fig. 1(A). The shift in retention time from 2.45 min to 2.26 min across the injection series correlates with the degree of alkali metal adducts (Fig. 1(A)) and some level of peak shape deterioration is observed (Fig. 1(C)). This observation confirms the previous conclusion that elevated levels of salts in the mobile phase can interfere with the charged‐based ion‐pairing retention mechanism. This is of particular concern for high‐throughput methods such as identity and purity screening assays that rely on short chromatographic runs where the gradients are optimized with minimal retention of the oligonucleotides.

When comparing the data shown in Fig. 3 to the 4^th^ injection of the trending data shown in Fig. 1(A) (spectral abundance 90.8%), which spatially represents the same amount of experimental time or column volume (CV) of mobile phase passed over the column (46 CV), potassium adducts were observed at trace levels (<1%) in the deconvoluted MS spectrum with sodium adducts accounting for the remaining 8.3% of the spectral abundance. Interestingly, when compared to the 30^th^ injection of Fig. 1(A) data, which equals the number of injections as the current experiment but represents a 8‐fold increase in column volumes (347 CV) of mobile phase passed over the column, spectral abundance was reduced to 43.1%, with multiple adducts of sodium and potassium observed. The current experiment repeatedly exposed the fluidic path between the injector and detector to an artificially elevated level of KCl normally not present in the mobile phase in less CVs then the data observed in Fig. 1(A) with only a marginal increase in adduct formation. This suggests the column is resistant to non‐specific adsorption of alkali metal salts. The decreasing shift in retention time in the borosilicate experiment (Fig. 1(A)), which was observed to be opposite of the sample purity experiment (Fig. 2(A)), indicates the source of salt adducts is most likely a mobile phase contaminant that is being ‘scavenged’ by a system component(s) or the column over time and is interfering with the ion‐pairing retention mechanism, thus contributing to increased adduct formation and retention time drift.

### Mobile phase solvents/reagents

From the previous experiments it was observed that the number of CVs passed through the fluidic path influenced the formation of metal adducts in oligonucleotide separations. To further investigate this phenomenon the 10 minute method previously used for the borosilicate analysis was modified to include the conditioning step at the end of the gradient to systematically increase the number of CVs the fluidic path is exposed to at initial mobile phase conditions. The system was cleaned of residual alkali metal salts prior to each run in the experiment. For consistency with the borosilicate experiment, the ssRNA (strand 1) was used for this experiment and prepared at the same concentration as before in the Optima series grade water.

**Figure 4:**
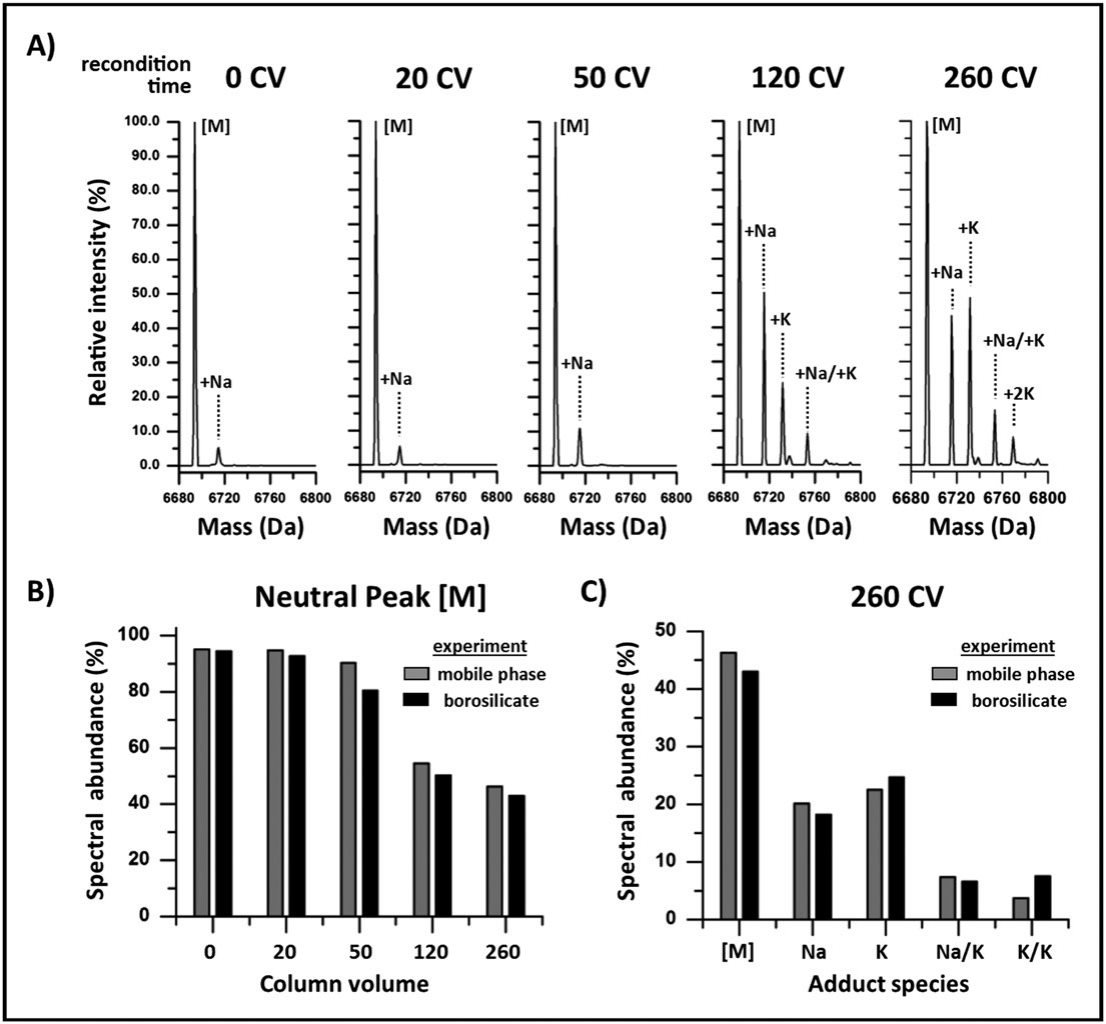
Mobile phase purity. Deconvoluted spectrum of the ssRNA strand 1 after flowing at initial conditions over a time period equal to 0, 20, 50, 120, and 260 column volumes (CVs). (B) Comparison of spectral abundance for the neutral peak [M] at equivalent CVs between the mobile phase and borosilicate experimental data set. (C) Spectral abundance adduct distribution profile between the mobile phase and borosilicate experiments for the 260 CV data set.

As shown in Fig. 4(A), adduct formation was observed to increase with CVs flowed through the system. The relative intensity of the sodium adduct exhibited the same trending behavior (10.8% vs 8.5%) when comparing the 50 CV experiment (Fig. 4(A)) to the 30 injection experiment shown in Fig. 3, which represents 39 CVs of mobile phase flowed through the system. This observation further corroborates the notion that the mobile phase is a contributing factor to adduct formation. Using the spectral abundance of neutral peak [M], a comparison of the borosilicate experiments (Fig. 1(A)) within 10 CVs of the current experiment were conducted. As shown in Fig. 4(B), the spectral abundance of the neutral peak [M] in the current mobile phase experiment exhibited nearly identical trending behavior as the borosilicate experiments. Closer inspection of the 260 CV data set as shown in Fig. 4(C) indicates almost identical distribution profiles of observed adduct species between the experimental data sets. The good agreement in adduct distribution and intensity in the current experiment with the borosilicate experiment indicates alkali metal impurities present in the mobile phase are the main LC contributing factor to adduct formation in oligonucleotide separations in the current study. The gradual increase in adduct abundance over time as observed in the mobile phase and borosilicate experiments suggest that the concentrations of impurities are at trace levels either in the solvent used in the mobile phase preparation or in the IP reagents themselves.

**Figure 5:**
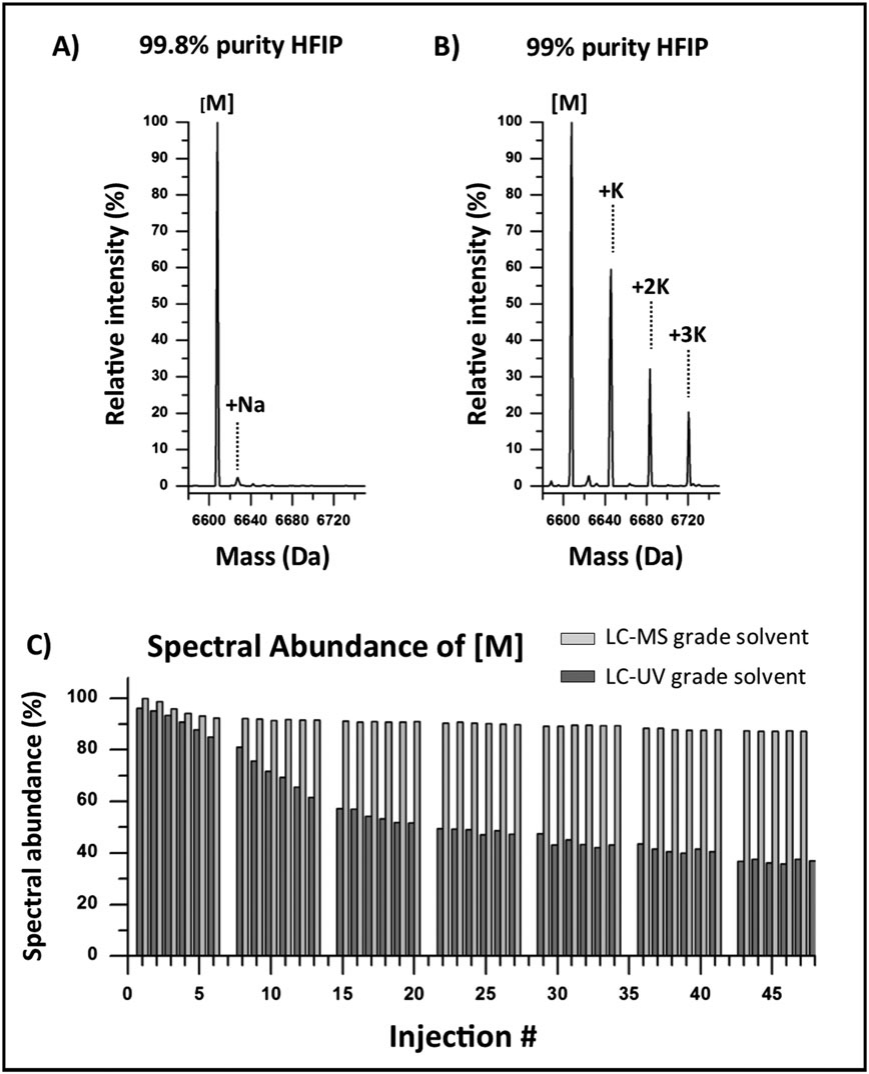
Reagent and solvent purity. Deconvoluted spectrum of the first separation of an ssRNA sample after removal of residual alkali metal salts using (A) 99.8% purity HFIP and (B) 99% purity HFIP. Sodium adducts were observed to have similar relative amounts in both IP reagents whereas significant potassium adduct(s) were observed in the first injection using the 99% purity IP reagent. (C) Spectral abundance was monitored for the deconvoluted parent peak [M] of the ssRNA (strand 1) over the course of an 8‐h injection series using LC/MS‐grade solvent and compared against the previous borosilicate experiment which used LC‐UV‐grade solvents. Both solvent systems were prepared with the 99.8% purity HFIP. Spectral abundance was observed to drop 13% and 57% for the LC/MS‐ and LC‐UV‐grade solvents, respectively, over the 8‐h injection series.

To elucidate the source of contamination, reagent and solvent samples were sent to an analytical service labs (VHG Labs, Manchester, NH, USA) for trace metal determination. As shown in Table 2, trace metal results using ICP‐MS analysis determined metal concentrations were below 0.5 ppm in the LC‐UV‐grade solvents as well as the IP reagents (Sigma TEA ≥99.5% purity and Fluka HFIP ≥99.8% purity) used in the previous tests. Trace level impurities of alkali metal salts are generally not a concern in routine UV‐based analyses; however, as the data has shown, trace level impurities can significantly impact spectral quality of sensitive LC/ESI‐MS‐based detection methods. Due to reagent matrix effects, assay methodology was limited to an limit of detection (LOD) of 0.5 ppm, thus requiring an alternative approach for further elucidation of the adduct contributing reagent. Comparisons of HFIP assayed with different purity (Sigma, ≥99% purity vs Fluka, ≥99.8% purity) were examined using the 10‐min high‐throughput method. As shown in Fig. 5(A) the ≥99.8% purity HFIP, which was used for the previous experiments and is rated for LC/MS use, resulted in a deconvoluted spectrum of the ssRNA with minimal adduct formation and was consistent with previous experiments. In contrast, the same experiment using the ≥99% purity HFIP resulted in primarily single, double, and triple adducts of potassium, as shown in Fig. 5(B), indicating a significant amount of potassium is present in the lower purity HFIP, which was assayed at a concentration of 46 ppm as shown in Table 2. TEA from a different manufacturer (Fluka) rated with the same purity (≥99.5%) was also investigated with similar methodology using the 99.8% MS‐grade HFIP as a buffer. Initial runs were similar with a single sodium adduct of relative intensity below 5% being observed which were consistent with previous baseline experiments. It should be noted that purity of reagent does not necessarily correlate with the observation of adducts (alkali salt concentration) as the reagents were from different vendors with different assay criteria.

**Table 2:** Reagent metal analysis. Trace metal results using ICP‐MS analysis for Na and K determined metal concentrations were below 0.5 PPM in all samples with the exception of the 99% purity HFIP, which was determined to have potassium present at a concentration of 46 PPM

Reagent	Na (μg/g)	K (μg/g)
UHPLC‐UV grade water	<0.5	<0.5
UHPLC‐UV grade methanol	<0.5	<0.5
LC–MS grade TEA (≥99.5% purity)	<0.5	<0.5
HFIP (≥99.8% purity)	<0.5	<0.5
GC‐grade TEA (≥99.5% purity)	<0.5	<0.5
HFIP (≥99.0% purity)	<0.5	46

To evaluate the mobile phase solvents, LC/MS‐grade solvents (CHROMASOLV series) were used to prepare the mobile phases using the IP reagents from the previous experiments (Sigma ≥99.5% TEA, Fluka ≥99.8% HFIP). Using the same experimental settings as the borosilicate experiment, spectral abundance for the neutral peak [M] was monitored over an 8‐h injection series. As shown in Fig. 5(C) spectral abundance of the neutral peak was preserved longer using the LC/MS‐grade solvents with only a 13% loss to adducts compared to 57% when using the LC‐UV‐grade solvents. These combined experiments indicate the purity of reagents and solvents significantly impacts the formation of adducts in oligonucleotide analyses, where the highest purity should be used for optimal system performance and improved assay robustness.^50^ With the main source of alkali metal adducts identified as the mobile phase, a mitigation strategy can be developed to reduce LC instrument contributions to metal adducts in oligonucleotide separations.

### Adduct mitigation strategy

The experimental data offers several insights into alkali metal salt adduct formation in oligonucleotide separations. It was determined trace‐level impurities (<0.5 ppm) present in the mobile phase act as a point‐source of alkali metal salts that are electrostatically adsorbed to non‐specific sites in the fluidic path that exhibit an affinity towards alkali ions based on the Hoffmeister series. Trending data shown in the borosilicate experiments (Fig. 1(A)) characteristically resemble a breakthrough curve where increased adduct formation to the polyanionic backbone of the oligonucleotide occurs gradually as available ions in solution increase.

Additional evidence supporting that the non‐specific adsorption mechanism is electrostatically driven can be observed in the higher spectral abundance of the single potassium adduct species, as shown in Figs. 1(E) and Fig. 4(C), which exhibits a higher affinity to negatively charged sites in comparison to the lower abundant single sodium adduct species. Furthermore, as shown in Fig. 4(B), the mobile phase experimental data represents 1 injection performed after flowing a set number of CVs with the reconditioning step performed once at an organic composition of 50%. In contrast, the data sets in the borosilicate experiment, which represent a similar number of CVs, were preceded by multiple injections using the same reconditioning step of 50% organic. However, the spectral abundances in the borosilicate experiments are only marginally lower despite being exposed to higher concentrations of organic solvent with higher frequency, suggesting the reconditioning step performed with higher organic composition (50% B) has negligible impact on reducing adduct formation over time. Interestingly, conventional LC‐based methods do not incorporate techniques to displace or desorb electrostatically adsorbed cations on fluidic path surfaces.

Current practices in LC/MS‐based oligonucleotide separations use IP mobile phases prepared at neutral or slightly basic pH. The non‐specific adsorption observed in this study, which exhibits behavior analogous to a cation‐exchange surface, requires exposure to solutions containing low pH (acidic) to displace adsorbed alkali ions or to ‘regenerate’ adsorption sites. Experimental evidence supporting this was demonstrated through the application of a cleaning protocol using phosphoric acid to acquire a baseline performance spectrum with minimal adducts (e.g. Fig. 1(D), injection 1). This knowledge, combined with the experimental evidence, suggests a method that incorporates a low pH mobile phase can be used to regenerate non‐specific adsorption sites and maintain MS compatibility.

To test this hypothesis, the system was exposed to trace salts by flowing eluent using isocratic conditions and evaluated periodically with same gradient as before (borosilicate experiment). Deconvolution results of the oligonucleotide peaks of the ssRNA strand 1 and strand 2 were used to confirm LC system salt contamination (data not shown). A low pH reconditioning method was implemented with MP C prepared as 0.2% formic acid in water (LC‐UV grade) and MP D prepared as neat methanol (LC‐UV grade). The 10‐min method from the borosilicate experiment was modified to switch to 50% MP C and 50% MP D for 1 min proceeding the separation gradient and then returned to initial method conditions for column reconditioning. A water blank using this low pH reconditioning method was performed prior to separation of the ssRNA samples to regenerate the fluidic path surface by displacing non‐specifically adsorbed cations. After surface regeneration, the ssRNA samples were separated using the low pH reconditioning method. Adduct formation was reduced to a single adduct of sodium (≤5.0%) even after a lengthy exposure of the system (≥5 h) to buffers containing trace salts.

**Figure 6:**
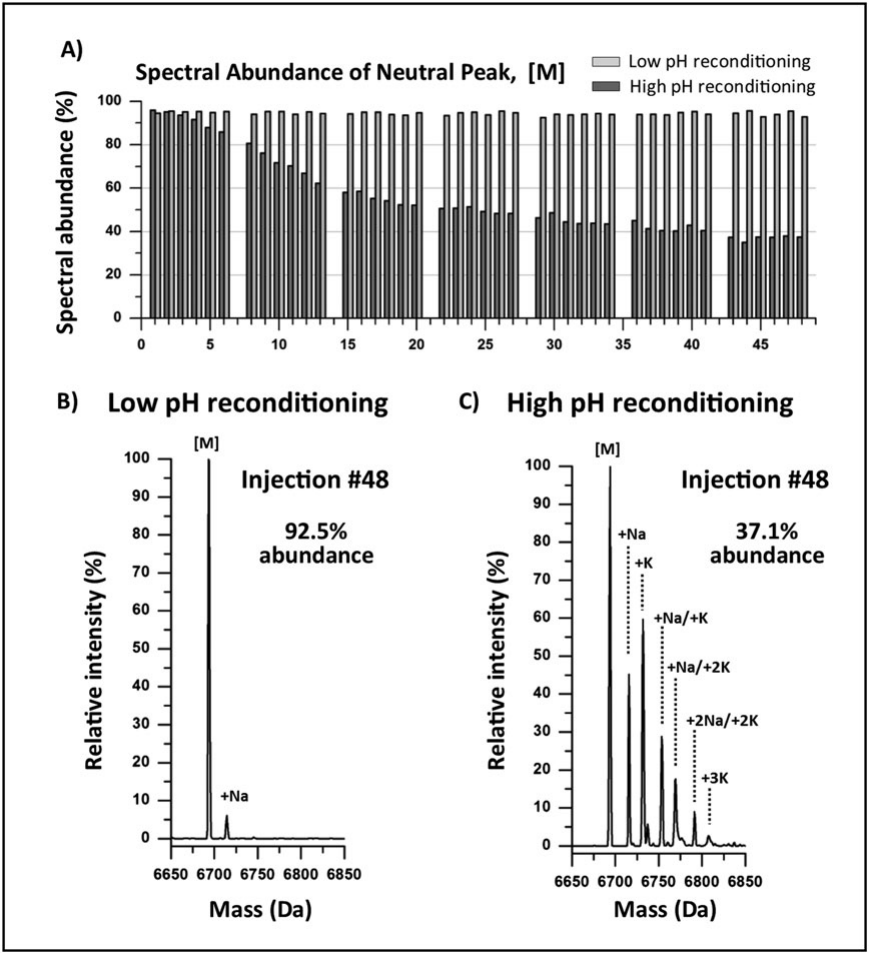
Comparison of spectral abundance of the neutral peak [M] for low and high pH reconditioning methods. (A) Spectral abundance for the deconvoluted neutral peak [M] was maintained above 92.5% using the low pH reconditioning step across an 8‐h injection series with an average spectral abundance of 94.5% (R.S.D. 0.8%). A 2‐fold increase in sensitivity was observed using (B) the low pH reconditioning method (92.5% abundance) when compared to (C) the high pH reconditioning method (37.1% abundance) for the neutral peak [M] across the 8‐h injection series.

Long‐term stability of the low pH reconditioning method to reduce alkali metal salt adducts in oligonucleotide separations was evaluated using the same experimental design as the borosilicate experiments using the low pH reconditioning method in lieu of the high pH reconditioning method with the LC‐UV solvents. Figure 6(A) shows that the spectral abundance for the neutral peak [M] was maintained above 92.5% across the 8‐h injection series when using the low pH reconditioning method with an average spectral abundance of 94.5% (R.S.D. 0.8%). The high recovery of spectral abundance using the proposed low pH reconditioning method resulted in over a 2‐fold increase in MS sensitivity when comparing spectral abundance (92.5% vs 37.1%). In addition, spectral complexity was reduced significantly with only a single adduct form being observed (Fig. 6(B)) for the low pH reconditioning method in comparison to multiple adducts (Fig. 6(C)) for the high pH reconditioning method across the 8‐h injection series. Retention time average was calculated at 2.44 min with an R.S.D. of 0.57% using the low pH reconditioning step; a testament to the robustness of the method. The same experiment was repeated using the LC/MS‐grade solvents in consideration for LC/MS‐based oligonucleotide assays that require even a higher degree of sensitivity such as toxicological profiles and metabolite studies.^14^, ^23^ The results indicated spectral abundance was maintained at 100% for the neutral peak with an average retention time of 2.45 min (R.S.D. = 0.59%) across the 8‐h injection series when using LC/MS‐grade solvents and reagents (data not shown). These observations demonstrate that the low pH reconditioning method effectively and consistently displaces non‐specifically adsorbed cations on surfaces throughout the LC fluidic path over multiple runs using an MS‐compatible acid with minimal impact on productivity.

With a successful method in place for mitigation of alkali metal adducts, an attempt to localize the adsorption site was made. Similar to before, salt exposure levels were increased using the LC‐UV‐grade mobile phases. Once salt contamination was confirmed the column was removed and cleaned with a 0.1% FA solution on a different system with an identical configuration that was previously cleaned. The column was then placed back in‐line on the contaminated system and the 10‐min high‐pH reconditioning method was performed. Adducts were observed to still be present at significant levels confirming previous observations the column has lower non‐specific adsorption of alkali metal salts relative to the fluidic path.

The mixer and plumbing before and after the mixer were tested in a similar fashion with the low pH reconditioning method being performed after disconnecting the fluidic path at each junction and flowing directly to waste. While relative spectral abundance fluctuated, baseline performance could not be achieved until all parts were in‐line and exposed to the low pH reconditioning method. The experiments indicate non‐specific adsorption occurs in the fluidic path; however, localization may not be possible as the adsorption process may be an additive property across the system. Adsorbed ions at trace levels and their sensitivity to residual protons in the fluidic path from the low pH recondition method increase the challenge in identifying the relative contribution of each component. Despite not being able to conclusively localize the adsorption sites, the current low pH reconditioning method effectively and consistently displaces non‐specifically adsorbed alkali ions in the LC fluidic path improving assay robustness and sensitivity.

## Conclusions

Current strategies to mitigate adduct formation have spanned a diverse set of offline and online approaches that have addressed sample preparation including desalting procedures that incorporate hydrophobic or ion‐exchange resins, molecular weight cutoff filters, and solid‐phase extraction techniques.^10^, ^12^, ^39^, ^42^ Alternative approaches to reduce complicated sample preparation procedures include the use of additives such as metal chelators including CDTA and EDTA that act as cation scavengers or bases such as piperidine and TEA that suppress adduct formation via a displacement mechanisms.^38^, ^46^ These approaches, while effective in reducing adduct formation, neglect to address LC instrument contributions to metal salt adducts, a challenging task considering the pervasive nature of alkali metal salts in LC separations.^28^, ^29^ The current study systematically evaluated common LC components in LC/ESI‐MS configurations used in oligonucleotide analysis to provide insight and strategies to mitigate metal adduct contributions from LC instrumentation.

The mobile phases used in this study were identified as the main contributing factor to metal adduct formation in oligonucleotide separations, and to a lesser degree the IP reagent. MS‐grade reagents demonstrated the least amount of adduct formation in the current study, a finding not entirely unexpected when using sensitive MS‐based detection methods. Interestingly, adduct intensity and abundance were observed to increase over time despite precautions such as preparing fresh solvents prior to analysis, using dedicated preparation glassware, and incorporating plastic‐based alternatives into the LC configuration. These observations combined with the fact the LC system could routinely be brought back to baseline performance with minimal adducts using a low pH cleaning protocol indicated non‐specific adsorption sites located throughout the LC fluidic path perpetuate adduct formation in oligonucleotide analyses.

The current study demonstrates that the presence of trace alkali metal salts can significantly diminish spectral quality in IP‐RPLC/MS‐based analyses of oligonucleotides. The intrinsic electrostatic attraction of metal cations with the polyanionic backbone of oligonucleotides combined with method conditions that favor adduct formation make MS‐based methods of oligonucleotides challenging. The current study addresses LC instrument contributions to metal salt adducts and provides a method for their mitigation. Implementation of a short low pH reconditioning step effectively displaces trace metal salts non‐specifically adsorbed to surfaces in the LC fluidic path. The proposed method offers the ability to rapidly regenerate adsorption sites with minimal impact on productivity while retaining assay sensitivity afforded by MS detection with reduced adduct formation, assay attributes highly desirable in the analysis of therapeutic oligonucleotides to ensure product safety, efficacy, and stability. Future studies will examine the applicability of the proposed method to a broader set of IP‐RPLC reagents and a more diverse panel of therapeutic oligonucleotides as well as its impact on chromatographic efficiency.
